# Parenthood, information and support on the internet. A literature review of research on parents and professionals online

**DOI:** 10.1186/1471-2296-10-34

**Published:** 2009-05-18

**Authors:** Lars Plantin, Kristian Daneback

**Affiliations:** 1 Department of Social Work, Faculty of Health and Society, Malmö University, Sweden; 2 Department of Social Work, University of Gothenburg, Sweden

## Abstract

**Background:**

The aim of this article was to address questions on how parents use the internet to find information and support regarding children, health and family life. Another aim was to find out how professionals use the internet to provide support and information to parents. This was done by a literature review.

**Methods:**

Articles were searched for in five databases with a search strategy called "building block" approach.

**Results:**

The review showed that the majority of today's parents search for both information and social support on the internet. However, there are considerable differences due to gender, age and socio-economic differences. First time middle class mothers aged 30–35 are most active in looking up health and parent information on the internet. In the same time, several studies report diminishing class differences on parent web sites. An important reason to the increasing number of parents who turn to the internet for information and interaction has shown to be the weakened support many of today's parents experience from their own parents, relatives and friends. Professionals have recognized the parents' great interest for going online and offer both information and support on the net.

**Conclusion:**

Many benefits are reported, for example the possibility to reach out to a wider audience and to increase access to organisations without an increase in costs. Other benefits include the possibility for parents to remain anonymous in their contacts with professionals and that parents' perceived need for information can be effectively met around the clock. Interventions for wider groups of parents, such as parent training on the net, are still very rare and more research is needed to evaluate different types of interventions on the net. However, most studies were empirical and lacked theoretical frameworks which leave questions on how we can more fully understand this phenomenon unanswered.

## Background

Today the possibilities to find information on children, health and parenthood on the internet are immense. When entering the term "parent and health" on the search engine Google it renders more than 90 million hits. If the search term is narrowed to the combination "parent, health and children" more than 50 million hits appear (as of 2009-05-12). Thus, parents are offered huge resources of information on topics ranging from children's diseases, deliveries and breast-feeding to more interactive web pages displaying ordinary problems in every day life, such as sore breasts, the upbringing of children or marital conflicts [1]. Statistical analyses of how different groups act and navigate on the internet have shown that many parents use the net as an important source for information. For example, market-research from the online network company Yahoo! showed that 86% of new parents-to-be used the internet to search for information on pregnancy and more than half of them claimed this to influence and simplify their lives [2]. But what do we know from the research on this relatively new phenomenon and what has been the scope of the research in the last decade?

In a prior study we investigated the *who*, *when*, *where*, *how *and, to some extent, *what *in the field of parenthood and the internet [3]. In addition, our aim was to identify dominating themes and trends in the existing body of research. This study, which was based on a systematic search for literature in 5 different databases, showed that the bulk of the articles were published after the year 2000 and that most studies were conducted in North America. The majority of articles were also empirical and conducted within the medical sciences. The analysis showed a fragmented field of research comprising few areas. Most studies focused on professionals' use of the internet in terms of education, information or interventions directed to parents, but some also discussed parents' information seeking on the net. It was concluded that in relation to the popularity of the internet among parents and professionals, relatively few studies have been conducted so far.

But, what do we know more specifically about parents' activities on the internet? To what extent do they use the internet to search for information about parenthood and children and how do they describe the benefits or limitations? What topics do they search for and how do they experience the social support on the internet? Overall, what are the advantages and disadvantages of using the internet in finding information and support about parenthood and children? The purpose of this study is to investigate these questions more closely and also to focus on how parents use the internet to find information and support regarding children, health and family life.

## Methods

Literature reviews can have different focuses and be conducted, methodologically, in many different ways. Some reviews claim to be "fully systematic" or "a meta analysis" with a strong focus on quality assessment of the selected research. Others are "traditional reviews" or "scope oriented" and more focused on the research findings themselves. This literature review can be related to the latter category, scope oriented, as it mainly focuses on what we know about "parenthood and internet"; thus it does not discuss the quality of the included studies [4].

### Search strategy

The literature review was based on a search strategy called the "building block" approach, a widely used search strategy that builds on similar and related terms combined in blocks [5]. Five databases were considered relevant for this topic. These were PubMed, ERIC, PsycINFO, Sociological Abstracts, and Social Services Abstracts. The databases were searched from inception until September 14, 2007, and the search was limited to human subjects, English and abstracts in PubMed and to peer-reviewed articles in English in the database platform CSA.

The search strategy was to build two blocks that would include terms (descriptors/MeSH terms) related to the internet and parenthood respectively and to combine the two blocks to capture the most relevant articles.

We included all terms that were related to parenthood in each database to build the first block. We then repeated this step for terms related to the internet to build the second block. The third block was simply a combination of all parenthood related terms and all internet related terms. Consequently, at least one term in the first block had to be matched with at least one term in the second block for an article to be considered relevant in this search. In the first and the second block terms were separated by OR and in the third block by AND.

### Inclusion and exclusion criteria

All of the matching references from the literature search were analyzed by the researchers to assess their relevance according to the topic parenthood and the internet.

The criteria for inclusion were that the articles had to be in English and peer-reviewed or, in the case of PubMed, have an abstract. They also had to focus on parenthood and the internet. We defined parenthood and the internet as when parents use the internet to seek support and information about pregnancy, babies/children and parenthood. By contrast, our definition also included professionals using the internet to reach parents or parents-to-be with information on web sites or direct interventions such as online counselling, support groups in web chat rooms or educational programs.

The criteria for exclusion were if the references were editorials or not focusing on our above mentioned definition of parenthood and the internet.

### Results of the database searches

The database searches yielded a total of 484 articles, the vast majority were found in PubMed. Of these articles, 109 articles were included by the researchers as relevant to the topic of parenthood and internet. Again, the majority of the articles were sourced from PubMed. In addition, we found that 15 articles were indexed in multiple databases, thus the actual number of relevant articles was lower, 94 articles in all.

A more detailed presentation of the method has been published elsewhere [3].

## Results and discussion

### Parents go online – needs and motives

We already know that parents and parents-to-be consume a vast amount of information about their own and their children's health and development [6]. This is done, for example, via television, magazines and books and this demand is reflected in the large array of baby-literature, lifestyle magazines based around parenthood and TV-programs such as Baby-boom or Super Nanny, that discuss child-rearing and the parenthood. According to many theorists and researchers, the underlying cause of this trend can be traced to the changing circumstances of the parenthood under post-modernism and an increased risk-awareness coupled with a reduction of support from parents' immediate friends and family [7]. O'Connor and Madge point to the fact that women traditionally have turned to their own mothers for support in their new parental role, but the increased mobilisation of the population has made close maternal-support more difficult to realize [8]. Mothers have, so to say, become more isolated and have, to a great extent, lost the daily support that they previously received from their families and other close relations [9]. Many women also highlight another form of problem coupled to the fast pace-of-change in modern society, namely that the information that they receive from their own mothers or elderly relations is 'out-of-date'. Their experiences are not valid any more [8]. Instead, parents are forced to trust the information they receive from various experts, in books, in parent magazines and on TV-shows et cetera.

At the same time, many researchers point to the fact that today's parents are no longer satisfied with simple descriptions of parenthood, but instead require more experience-based information, in other words, knowledge that conveys the experiences of others in similar situations as themselves. For example, how does it actually feel giving birth, and how do other parents cope with the challenges of breast-feeding? In parallel to this development, many researchers in family sociology also point to the fact that 'family' as a form of living and as a collective-noun is also changing over time [10,11]. Bäck-Wiklund discusses, for example, how the post-modern family can no longer be described as a closed social unit, but rather as a network of close relationships [12]. Globalisation, an increasing divorce-rate and increasing numbers of re-built families or stepfamilies, have contributed to the trend of an increased number of family members and a wider geographic spread of close family members. It is here that modern information technology has played an increasingly important role in terms of how it can facilitate the maintenance of emotional ties and the communication of 'nearness' that are so important in day-to-day relations. Today's parents are more accustomed to finding companionship and communication closeness via the internet. Researchers also point out that a further fact influencing modern parents' need for information may be due to the increasingly shorter stay in hospital after giving birth. Prior studies highlight the importance of both support and information in order to have the parents, who have just returned home with their newborn baby, feel secure in their new roles [13,14]. Otherwise, there is a risk that many parents, especially first-time parents, are left alone with their questions and in need of information [15].

Bylund's study of a website where parents, mothers in particular, post their experiences of delivery, shows that there are primarily four main driving forces which dominate the need to share birthing-stories on the internet; firstly that they have an entertainment value for the individual concerned, secondly that they create a possibility of making contact with other women/mothers who have had similar experiences, thirdly that childbirth is a 'rite-of-passage' and finally that it is an important way to come to terms with the traumatic experience of childbirth [16]. Similar driving-forces, especially the need for positive affirmation and the chance to measure or share experiences of one's parenthood with others, have been confirmed in other studies. Bernhardt and Felter's interviews with mothers, who were online for health-related information, reveal that they primarily did so to learn more about fetal and child development, to find out more about diagnosing and treating specific paediatric health conditions and to find experience-based support for a variety of parental issues [17].

Similarly, many of the women in Madge and O'Connor's study reported that the social support from other mothers relieved or neutralised the feeling of isolation and a shrinking life-world that can be experienced during maternity leave [18].

To use the internet as an information source or to establish contact with others in similar situations is of particular importance for parents whose children are suffering from different varieties of illnesses or whose children are handicapped. Unexpected occurrences can also trigger a strong desire for support and information. For example, in their study of parents with premature babies, Brazy, Andersson, Becker and Becker state that the respondents "made a transition from being passive recipients of information to actively seeking it", especially during the time that the child remained in hospital care [[19], p.41]. This is also confirmed in studies of parents whose children are suffering from mental illness, neurological disorders or autism, showing that parents value highly the social and emotional support they receive via the internet [20-23].

Lawton, Roberts and Gibb claim that parents of children suffering from a chronic skin disease not only wanted to find professional experts' advice on the internet, but also wished to share their own experience-based expertise that they had built-up over time [24]. There is, thus, a substantial need for many parents to both receive and share information about children and parenthood, which, to quote O'Connor and Madge, probably suggests:

"that the internet does offer an important source of advice and support for groups such as new mothers, in particular by providing a 'safe', non-judgmental forum in which to share experiences. However, this does not replace face-to-face communication or more traditional support mechanisms such as family, friends, and health careers; rather it serves to supplement these by providing an additional resource" [[8], p.351].

### Searching for information and support – parent's online behaviour

In the light of the developments mentioned above, it is not difficult to understand parents increased use of information provided by the internet regarding children, health and parenthood. A report on American parents' online behaviours show, for example, that close to 70% of parents had gone online to look for health-related and medical information [25]. A similar market-research study by the online network company Yahoo! showed that 86% of parents-to-be use the internet to search for information about pregnancy [2]. This is also confirmed in Bernhardt and Felter's qualitative study of how pregnant mothers and mothers of young children seek and process online paediatric information [17]. It reports that a majority of mothers used the internet in their search for health-related information. For younger first-time mothers, the internet is often the primary source of information, whilst older first-time parents and parents with older children either used books or direct doctor contact as their primary source of information, before turning to the internet.

The majority of parents who use the internet report that they find health-related information online primarily by using search engines. Others find the information they require either via recommendations from friends or through adverts in various parental magazines [26].

The search for health-related and medical information is often the primary reason for men and women to start using parental web sites [27]. There has been a phenomenal increase in the number of such sites and today many sites are entirely dedicated to parents and parents-to-be. These sites often contain a mix of activities and offers, ranging from commercial products to health-related information and the possibility to establish social contacts. Bernhardt and Felter describe parent web sites as places where parents can "shop, socialize, and research a wide range of topics" [17]. The British parental web site "Netmums" serves as a typical example of this virtual environment for parents. It provides links to web pages about everything from morning sickness remedies, information on vaccines, coughs and colds, to the Netmums own "coffeehouse" where members can chat with other parents and get support or advice on anything to do with being a parent – "from little gripes to big issues" [28]. Additionally, there are also more specialised chat rooms and online groups for parents suffering from postnatal depression. These interactive web sites, where parents quickly at any time of the day and night can get experience-based information from many people in situations similar to their own, are valued by many parents on the internet [1,8,29-31].

This is evidenced by the steadily increasing number of visits to parental web sites. Russell point out that in 2006, Netmums had around 140,000 members [32]. Today the number of members has doubled to over 550,000 (19-03-2009). It is possible to see a similar development on the Swedish parental website FöräldraNätet (English translation: The Parenting Network). In 2004 the researchers Sarkadi and Bremberg reported that the site had between 50,000 and 60,000 unique visitors per month [33]. According to the Swedish Advertising Association, the corresponding figure for the site today, in March 2009, is closer to 242,000 unique visitors per month.

Additionally the website reports that around 30,000 comments are registered each day in their chat room. Similar results concerning the visitor frequency for parenting web sites are confirmed by Madge and O'Connor [18]. In their online survey distributed through the web site  they found that more than two thirds (67%) of the respondents visited the parenting web site at least once a week. A small group of parents, 8% of the respondents, visited the web site on a daily basis. Most of the parents in the study browsed the web site for an hour or less each time.

Bernhardt and Felter report that parenting web sites are generally regarded as easily accessible while more academic or non-commercial web sites with information on health, children and parenting often are experienced as less attractive and harder to use [17]. Pure commercial web sites, such as the corporate web sites of baby-porridge or baby-diaper companies, are the least visited web sites.

Thus, prior research draws a clear picture of both an increasing variety of web sites for parents and an increasing number of parents active on the internet. But what do we know more precisely about the parents online? Who are they and what motivates their search for information and support on the internet?

### Going online – a digital divide?

Today there is an increased professional, research-based and commercial interest in finding out more about parents who are active on the internet. One of the underlying reasons is the desire to target both information and products to the different parental groups more precisely.

The demographic profile of the average internet-using parent is that of a young, white, middle class woman, who uses the internet mostly to search for health information and to visit parental web sites [25,34]. Although user demographics vacillate over time, the vast majority (85–95%) of parental internet users are women [18,33]. Similar figures apply when looking at parental internet users who are searching for health-related information, where 80% of women have at one time or another used the internet to search for health-related information [34]. Broadly speaking, it is possible to say that when it comes to searching for information about health and parenting, women's online behaviour confirms their offline behaviour as they most often take the main responsibility for the hands-on healthcare of the family [26].

The mean age of these parents is less than 35 years. In Madge and O'Connor's study on the British parenting web site 'Babyworld', 76% of the visitors were under 35 years [18]. Similar figures appeared in Sarkadi and Bremberg's study of a Swedish parenting website [33]. An important explanation to the relatively young age of the average parental website visitor is the fact that they are often first-time parents [33]. An Israeli study of an internet-based parental advice forum, reports almost all visitors to be women and that 62% of them were first-time parents [35]. Another explanation can be that younger parents are more accustomed to the daily use of the internet compared to older parents.

The main differences in parents' use of the internet can be found in disparities within income, ethnicity and education. This gap is referred to in literature as the 'digital divide', which states that internet use is more widely spread in more socio-economically advantaged groups [36]. For example, statistics on the American use of the internet show that only 29% of Latino adults have a broadband connection at home compared to almost 50% among white adults [37]. Similar differences are found in comparison with other ethnic groups as well. Brodie, Flournoy, Altman, Blendon, Benson, and Rosenbaum report, for example, that African-Americans had significantly less access to computers at home compared to white Americans [38]. The same applies to their access to the internet and their inclination to search for health and medical information. At the same time, the authors underline the fact that even if the gaps between African-Americans and white Americans are particularly pronounced in these areas they are largely a function of income and "tend to disappear once we compare blacks and whites at higher income levels" [[38], p. 257]. Similar results are shown in Cotten and Gupta's research, which demonstrates that larger societal factors such as income and education influence people's choice of where they look for health-related information [26]. Using the internet to find health-related information was more common for those with higher incomes, higher education and better health.

Social class is an important factor in our understanding of internet-usage and the search for health-related information, and this is just as valid for parents' usage. In their study of parents with a handicapped child, Blackburn, Read and Hughes claim that "a significant number of carers may not currently be internet users and that age, gender, socio-economic status and caring responsibilities shape the internet in particular ways" [[36], p. 201]. They draw the conclusion that, "it is important to address the digital divide among carers and to continue to develop other services and information systems to meet the needs of those who do not access the internet." But despite the fact that prior research shows that social inequalities affect parents' opportunities to search for information or to receive social support through the internet, it is by no means conclusive.

Dunham, Hurshman, Litwin, Gusella, Ellsworth and Dodd report in their study of a web-based project aimed at 42 single mothers that no differences in internet-usage could be attributed to either educational level or to social class [1]. Instead, it was possible to conclude that those most socially isolated with the fewest friends were the most prevalent internet users. There are similar results in studies of more 'general' parenting sites. Russell reports around 40% of the visitors at Netmums to be from families with lower incomes [32]. Sarkadi and Bremberg show in their Swedish research of a similar parenting site that, even if the respondents' educational level were slightly higher than the national average, 68% had income levels under the national average (some were on maternity leave, studying et cetera) [33]. In line with Dunham's study, they report factors such as living without a partner and having low educational and income levels to increase the need for support to these parents (mothers). It is possible that the increased availability of computers and the internet can be seen as an explanation to why more parents from less privileged socio-economic groups use internet-based parental sites in their search for support and information. In Sweden alone, internet availability for citizens 16–74 years old increased from 73% in 2003 to 84% in 2007 [39]. A similar tendency is seen in all the member states within the European Union [40]. The internet accessibility in EU increased 5% overall in 2007, but in some of the new membership countries, like Romania and Slovakia, it increased for example by 57% and 70% respectively.

The readability of the information one can find on the internet is another important factor when it comes to explaining differences according to socio-economic position in using the internet to seek health information. Several studies show that the readability level is often too high for the majority of people [35]. For example, examining 25 health web sites, Berland et al found that almost all of the sites required high school level or greater reading ability [41].

When parents go online, what kind of information do they look for? We have previously mentioned that they look for information on health and child development [17], but more specifically, what topics are discussed and what kind of information is sought after? Do parents trust the information they find? Do they rely on it?

### Topics and the quality of information

Health-related information during pregnancy is a topic of high interest for many soon-to-be parents and many seek information regarding fetal development, labor and delivery. To be more precise, Mankuta, Vinker, Shapira, Laufer and Shveiky claim that many soon-to-be parents search for information about pregnancy complications such as pre-eclampsia, pregnancy-related diabetes, fetal diagnostics and information regarding different forms of delivery [42]. They also report that parents of newborn babies are often less active in their search for information, probably as a direct result of the fact that they are preoccupied with the newborn baby and have less time to use the computer. But for those who did use the internet, their questions were on topics ranging from bleeding and other post-delivery complications, through to post-natal depression, stress and anxiety of first-time parents.

Other studies also demonstrate that parents of young children primarily seek information regarding the diagnosis and treatment of childhood illnesses [17]. In most cases this revolves around the need for complementary information, to get a second opinion, to complement the information already provided by their doctor or to confirm what they are already thinking [8]. Many parents also seek advice and confirmation regarding the upbringing of their children and seek reassurance that their children's behaviour is regarded as 'normal' [8,36,43]. In Sarkadi and Bremberg's Swedish study the results are the opposite [33]. There the majority of parents used parental web sites as their primary source for information and advice about children and parenthood. Corresponding results are shown in another similar study [17].

The questions and subjects of conversation in the interactive chat forum tend to be even more diversified. Dunham, Hurshman, Litwin, Gusella, Ellsworth and Dodd analyse the topics of the online conversations on a web site's public discussion forum [1]. They show that women discussed and sought support on a variety of day-to-day issues such as aching breasts, the need to find larger and better apartments, disputes over child care, advice on upbringing, parental conflicts, the best way to treat ear infections, allergies, sleeping problems or toothache, how best to handle the bureaucracy within social services or primary health care, how to use a breast pump or where to buy the cheapest nappies. The sheer variety of conversational topics discussed in the discussion forum was striking and led the researchers to conclude that "it is difficult to imagine any single community service providing, in such a timely manner, the diverse amount of information and support that these women exchanged on a daily basis" [[1], p. 299].

However, many studies report that health-related information available to parents on the internet can be misleading and occasionally, utterly wrong [44,45]. For example, in a survey of 41 parent-oriented web pages relating to taking care of fever-ridden children at home, Impicciatore, Pandolfini, Casella and Bonati indicate that only a few (four web pages) provided complete information according to the recommendations in the general guidelines [46]. The remaining web pages gave advice and recommendations that deviated to a varying degree from the recommendations stipulated in the general guidelines. In addition, Dornan and Oermann and Shaikh and Scott analyse large numbers of web sites containing information on breastfeeding and conclude that there is great variation in the details of the information and its practical usability [47,48].

Dornan and Oermann's research claim that only 7 out of 40 web sites matched all of the quality criteria in the model they had developed for testing the quality of information provided. In many cases these web sites are created and maintained by individuals or self-help groups with possibly fewer resources to enable them to uphold the quality of the information. The results from Hardwick and MacKenzie's study would point in this direction [49]. When analysing 19 web sites containing information about early onset miscarriage they found that the majority of web sites were maintained by either individuals or self-help groups and that the quality of the information was generally poor when compared to the guidelines stipulated by the Royal Collage of Obstetricians and Gynaecologists. In most cases the web sites lacked basic information and in some cases the information provided directly contravened the aforementioned guidelines.

Similarly, Ernst and Schmidt conclude in their study of web sites provided by 'medical herbalists' for pregnant women that "the advice offered was misleading at the best and dangerous at the worst" [[50], p. 190]. One of the most exhaustive studies of the quality of health-related information available on the internet is done by Eysenbach, Powell, Kuss and Sa [45]. The study comprises 79 separate studies, which in turn comprise the analyses of 5941 web sites and 1329 web pages. The conclusion reached in 70% of the studies is that quality is a problem on the web.

At the same time, one should be aware that in the majority of studies the quality of information is assessed from a medical point of view. Other perspectives, like the point of view of a patient or a lay person, would likely render a slightly different view of the 'quality' of the information [51].

Given this background, it is not surprising that many parents are sceptical of much information that they find on the internet [52]. According to Bernhardt and Felter parents use a wide variety of non-scientific verification methods when trying to ascertain the quality of the information on a given web site [17]. Typically they begin by trying to deduce the underlying motive of the web site where they find the information. This can be done, for example, by asking the question; is there someone who is making money from this information? They also try to ascertain whether the original source of the information is trustworthy, that is to say that the information comes from a trustworthy organisation or source. The most valued sources, as shown by other research, include links to governmental organisations, universities and volunteer organisations. In addition, they have a professional layout, understandable language and good references to other sources of information [45,47]. Moreover, parents weigh the information they find. This means that if the information found is corroborated by several independent web sites, the validity increases. Finally, many parents feel that the quality of the information increases if it is verified by other parents. However, one of the few observational studies available on this topic shows that many internet users most often do not check the motives and the "about us" section of the web sites [53]. Thus this indicates a gap between theory and practice on this matter.

O'Connor and Madge claim that many parents, especially first-time parents, feel generally positive about the information they find on the internet [8]. In many cases they find it easier to access and to be more up-to-date compared to the information provided by healthcare professionals, for example within maternity care. Some studies show that there are gender differences in the need to verify information found via the internet. For example, Cotten and Gupta mean that men are less concerned about the credibility of online health information than women [26].

In response to the fact that there is such a vast amount of misleading health-related information on the internet, there is a number of different organisations today that offer help to find health-related web sites with valid information. The "Health On the Net Foundation" is one example of an organisation that guides around half a million visitors each month to web sites with "useful and reliable online medical and health information" [54].

We are able to see that many parents and parents-to-be use the internet in order to find health-related information and to exchange experiences around pregnancy, children and daily life as a parent. Many parents choose the internet as their first point-of-reference over other sources of information including professionals in offline settings. The amount of information available, however, is vast and there is a considerable risk that the information is inaccurate and unreliable. In that case, how do professionals and support organisations that work with parents and parents-to-be use the opportunities provided by the internet? Do they target their information to parents via different web sites or do they use the internet to work directly with parents, individually or on a group basis? In the next section we will take a closer look at the research on how professionals and organisations use the internet in terms of education, information or interventions directed to parents.

### Directing information and interventions to parents on the internet

As we were able to conclude in a prior study, a large number of studies have evaluated the possibilities for institutions and organisations to reach parents with information or various interventions over the internet [3]. This development is partly due to the fact that today's parents are more active in their use of the internet as a source of information and partly due to the fact that research suggests the internet to be an effective medium for providing support to a large number of parents [47,55,56]. Also, to use the internet is considered by many institutions as a way to increase the efficiency of their support to parents, a way to reduce the pressure on their ordinary treatment centres and a way to reach out to sparsely populated areas as well as to be able to provide a more unilateral form of support for parents. In addition, the internet is shown to provide a good way to increase cooperation with parents and to reduce organisational expenses [43,57,58].

As most of the research within this area has been conducted within the medical sciences, most of the studies are clinical and linked to or carried out in hospital settings [3]. This is the reason why many of these studies focus on support to parents whose children suffer from diseases or are disabled. More specifically, these studies revolve around the development of support in the form of informative web sites comprising internet support groups for parents of children with, for example, brain injuries [59], cancer [52,60], deformities [61], neurological disorders [22], skin diseases [24] and asthma [62].

In addition, many studies also focus on more general information and support provided online to parents and parents-to-be. Some examples are maternal and child healthcare services that wish to provide information in the form of parental education [56,63], spread information about prenatal genetic counselling [64], C-sections [65], breast feeding [31] or paediatric clinics that wish to establish a web site with the purpose to help families cope with ordinary childhood diseases [66].

A majority of the intervention studies are based not only on the provision of information via web sites, but also on interactive support of parents via discussion fora and internet support groups. Then what experiences do professionals have of using the internet to provide support and how do parents respond to the support offered to them?

### Experiences and interventions on the internet

Despite the numerous possibilities of giving and receiving valuable support and information via the internet, some studies show that both professionals and parents also have negative experiences. Referring to the previous discussion on the digital divide, one of the primary problems can be that many parents simply do not have access to internet connected computers [36]. As a result, parents from more socio-economically disadvantaged groups are at risk to be excluded from internet-based information and support. Amongst professionals and parents who have access to the internet, the negative experiences often revolve around problems with usability, such as technical problems, incompatible hardware, access difficulties or lack of technical and management support [33,67].

In a similar vein, many professionals and parents lack interest in using the internet and lack confidence in their perceived skills to use the internet [30]. Kouri, Turunen, and Palomaki claim that there is a resistance to use the internet as a means to provide support to parents as employees feel that they do not possess the necessary skills [58]. A number of employees also doubt whether or not parents, specifically mothers, are interested in internet-based support instead of "more personal contacts" [[58], p. 181]. Their misgivings are to a certain extent supported by parents who, in Han and Belcher's study, complain about the lack of physical contact and proximity when partaking in support groups on the internet [60]. They also point out, in similarity to parents in a variety of other studies, the negative aspects including large volumes of e-mails, off-topic chatter and occasionally intensive quarrels in the discussion groups [33]. The fact that misunderstandings and quarrels occur easily over the internet is not surprising according to Scharer, as inexperienced users easily hit the caps lock button and "as capital letters are often considered as shouting, this may give unexpected consequences" [[20], p. 31].

The risk that people are rude, disrespectful or in one way or another disrupt communication is always there, but many studies of internet-based discussion groups also show the opposite to be true, that the groups report a marked absence of disruption. Instead it is possible to conclude that discussion group users follow a sort of 'netiquette', an internet etiquette, which contains both formal and informal rules governing respectful communication on the internet [8,29].

Possibly, this may be the reason many parents report overwhelmingly positive experiences of support through the internet [1,8,17,29-31,68,69]. For example, Baum reports that parents of children with special healthcare needs participating in an online support group "found more than expected in terms of insight and people to trust" [[70], p. 381]. Nearly 90% of the sample considered participating in a support group again as soon as possible. Sarkadi and Bremberg report that to chat on parent web sites is important for parents and provide them with valuable space for discussions [33]. The advantages, according to the respondents, of being able to search for information on the internet and being able to participate in interactive discussion fora, include accessibility, speed of feedback, anonymity and democratic discussion climate. Especially interesting to note is that both single parents and parents with a lower level of education also experience a strong sense of support from the parental web sites.

In addition, O'Connor and Madge mean that the internet is very important for those women who use it; "it played a central role in providing virtual social support, increasing the women's ability to cope with new parenthood and the shared experience it provided was important in the transition of identity to motherhood" [[8], p. 365]. As a consequence, only a few of the participants admitted to lie about themselves or their identity. However, it needs to be underlined that a majority of parents do not feel that virtual support can entirely replace face-to-face meetings, but instead that it should be regarded as a valuable compliment.

Nevertheless, many studies focus on communication in chat rooms or support groups on the internet and describe both the proximity and the strengths of the relationships formed there [29]. Among other things Duham, Hurshman, Litwin, Gusella, Ellsworth and Dodd report that "close personal relationships and a sense of community developed in this novel social environment" [[1], p. 282], which refers to an intervention aimed at single mothers where the organisers offered a computer-mediated social support network around parenting issues. Similarly, but possibly more conclusive, Capitulo's report from a support group for parents who had lost a child show that very close relationships and even a specific 'culture' developed within the group. They shared, Capitulo says, "virtual identities and created a community where they brought meaning to their perpetual losses". They also created a collective identity, 'Mothers of Angels' which created a strong sense of belonging and the message that "you'll never be alone" [[71], p. 305].

The support available via the internet has some negative aspects, but despite this the vast majority of users experience it as very positive. However, only few studies are able to claim whether or not the virtual interventions on the internet are more effective than interventions 'in real life'. This is due to the design of these studies where most have not used control groups. One of the notable exceptions, however, is Hudson, Campbell-Grossman, Fleck, Elek and Shipma who compare two different groups of fathers, one taking internet-based parental guidance classes and the other taking the standard parental guidance classes provided by the maternal and child healthcare services [56]. A total of 34 first-time fathers took part in the research, evenly divided between the two groups. The group of fathers partaking in the internet-based parental guidance classes used the website 'New Fathers Network' where they had access to a virtual library containing literature on children and parenthood. They also had access to a discussion forum where they could meet and interact with other parents. In addition, it was possible to e-mail their questions to a midwife. The study focuses on the fathers' competence and trust in their own parenting abilities and measured this both 4 weeks and 8 weeks after the birth of their child. The study reports more positive results for the group taking part in the internet-based parental guidance classes compared to the fathers in the control group. The fathers in the internet group reported a marked increase in both their competence and their trust in their own parenting abilities during the test period.

This is particularly interesting in comparison with the notion that regular maternal and child health services have experienced difficulties in reaching and interesting fathers-to-be [70,72]. It clearly points to the fact that one needs to look at alternative methods to reach fathers and break the traditional gender division in matters relating to reproductive health. To facilitate discussion groups and parental guidance classes on the internet may be good alternatives to the more traditional discussion groups common within maternal and child healthcare services. Maternal and child healthcare services have previously been criticised for leaving men at a disadvantage as women are more used to talking about pregnancy, birth and parenting [73].

Nevertheless, many of the parental web sites on the internet are still traditionally gender-biased and are more oriented towards mothers' needs. The parental web site Sarkadi and Bremberg refer to in their study is a typical example of this. Many of the fathers included in their study complain that they feel marginalised due to the discussion groups being dominated by women and centred on motherhood [33]. One conclusion is that more specific discussion fora are necessary to better cater for the specific needs of men/fathers. Rashly identify and criticise the fact that many web sites encompass a traditional gender bias towards mothers [27]. She specifically refers to commercial web sites, which at first appear to be progressive and strive for greater equality in the parental role, but which at closer inspection simply reproduce traditional gender roles and values. Virtual life, in this case, seems not to differ much from real life.

## Conclusion

As this review of literature shows, the majority of today's parents search for both information and social support on the internet [8,25]. Their questions cover many topics, from pregnancy and birth complications to child discipline and child illness. It is not only a desire for facts and information but an interest in more experience-based advice as well as interacting with other parents [1]. The reasons for this development are often related to the increase in accessibility to information through the internet as well as in the parents' use of the internet for communication. [25]. A number of articles indicate that parents of today experience diminished support from their own parents, relatives and friends and, consequently, turn to the internet for information and interaction [8,18]. However, most of the studies are empirical and lack theoretical frameworks which can help to understand this phenomenon of parents' increasing pursuit of information. How can we understand this phenomenon in parallel to other changes which occur in the late-modern society, for example increased individualisation, reflexivity and awareness of risks? So far, questions of this kind are rarely discussed in the body of research on parenthood and the internet. Moreover, there is a lack of longitudinal studies that focus on parents' internet use over time and in different phases of their parenthood.

At the same time the empirical knowledge-base of parents' use of the internet is quite comprehensive and detailed. It is known, for example, that women are more active than men in looking up health and parent information on the internet, and that the majority of visitors to web sites on parenthood are first-time parents in the age group 30–35 [33]. Many studies also discuss class and ethnicity from the concept of the digital divide which states that the internet is less used in more socio-economically disadvantaged groups and in certain ethnic groups [36,37]. However, most discussions of ethnicity are more about access to economic resources since differences due to ethnicity often tend to disappear at higher income levels [38]. In addition, many studies report diminished or negligible class differences among visitors to various web sites directed to parents. This means that huge class differences among participants on parental web sites cannot be automatically assumed, but observed as the field develops.

Due to the increasing access to the internet and the success in reaching out to a wider group of users, an increasing number of professionals have been encouraged to use the internet to disseminate information and to initiate discussions around parenthood and health. There are many benefits to be found and the possibility to reach out to a wider audience and to increase access to organisations without an increase in costs are examples of important arguments for using the internet presented in many studies. Other benefits include the possibility for parents to remain anonymous in their contacts with professionals and that parents' perceived need for information can be effectively met around the clock.

Research in this area, however, has been dominated by the medical sciences and subsequently the majority of direct interventions – and their evaluations – on the internet are first and foremost directed to parents with very specific needs. Interventions for wider groups of parents, such as parent training on the net, are still very rare and more research is needed to evaluate different types of interventions on the net. However, in a broader perspective, the interest for many parents to use the internet in their parenthood may also stimulate many professionals to find new possibilities provided by the internet in their work with parents and families.

## Competing interests

The authors declare that they have no competing interests.

## Authors' contributions

LP and KD designed the study, made the database searches, and analysed the retrieved articles. LP drafted the paper and KD contributed to later versions. Both authors read and approved the final manuscript.

## Pre-publication history

The pre-publication history for this paper can be accessed here:



## References

[B1] Dunham PJ, Hurshman A, Litwin E, Gusella J, Ellsworth C, Dodd P (1998). Computer-Mediated Social Support: Single Young Mothers as a Model System. American Journal of Community Psychology.

[B2] Yahoo (2005). Yahoo! Search marketing launches "Life Series" to explore relationships between major life events and internet search habits". Yahoo media relations.

[B3] Daneback K, Plantin L (2008). Research on Parenthood and the Internet: Themes and Trends. Cyberpsychology: Journal of Psychosocial Research on Cyberspace.

[B4] Arksey H, O'Malley L (2005). Scoping studies: towards a methodological framework. International Journal of Social Research Methodology.

[B5] Marchionini G (1995). Information seeking in electronic environments.

[B6] Hagström C (1999). Man blir pappa Föräldraskap och maskulinitet i förändring.

[B7] Beck-Gernsheim E (2002). Reinventing the family – In Search of New Lifestyles.

[B8] O'Connor H, Madge C (2004). 'My mum's thirty years out of date': The role of the Internet in the transition to motherhood. Community, Work & Family.

[B9] Drentea P, Moren-Cross JL (2005). Social capital and social support on the web: The case of an Internet mother site. Sociology of Health & Illness.

[B10] Roman C (2004). Familjen i det moderna Sociologiska sanningar och feministisk kritik.

[B11] Castells M (1997). The Power of Identity – The Information Age: Economy, Society and Culture.

[B12] Bäck-Wiklund M, Bäck-Wiklund M, Johansson T (2003). Familj och modernitet. Nätverksfamiljen.

[B13] Persson E, Dykes AK (2002). Parents' experience of early discharge from hospital after birth in Sweden. Midwifery.

[B14] Persson EK, Fridlund B, Dykes AK (2007). Parents' postnatal sense of security (PPSS): development of the PPSS instrument. Scandinavian Journal of Caring Science.

[B15] Lamp JM, Howard PA (1999). Guiding parents' use of the Internet for newborn education. The American Journal of Maternal Child Nursing.

[B16] Bylund CL (2005). Mothers' involvement in decision making during the birthing process: a quantitative analysis of women's online birth stories. Health Communication.

[B17] Bernhardt JM, Felter EM (2004). Online Pediatric Information Seeking Among Mothers of Young Children: Results From a Qualitative Study Using Focus Groups. Journal of Medical Internet Research.

[B18] Madge C, O'Connor H (2006). Parenting Gone Wired: Empowerment of New Mothers on the Internet?. Social & Cultural Geography.

[B19] Brazy JE, Anderson BM, Becker PT, Becker M (2001). How parents of premature infants gather information and obtain support. Neonatal Netw.

[B20] Scharer K (2005). An Internet Discussion Board for Parents of Mentally Ill Young Children. Journal of Child and Adolescent Psychiatric Nursing.

[B21] Scharer K (2005). Internet Social Support for Parents: The State of Science. Journal of Child and Adolescent Psychiatric Nursing.

[B22] Leonard H, Slack-Smith L, Phillips T, Richardson S, D'Orsogna L, Mulroy S (2004). How can the Internet help parents of children with rare neurologic disorders?. J Child Neurol.

[B23] Fleischmann A (2005). The Hero's Story and Autism. Grounded Theory Study of Websites for Parents of Children with Autism. Autism the International Journal of Research and Practice.

[B24] Lawton S, Roberts A, Gibb C (2005). Supporting the parents of children with atopic eczema. Br J Nurs.

[B25] Allen K, Rainie L (2002). Parents online. Report.

[B26] Cotten SR, Gupta SS (2004). Characteristics of online and offline health information seekers and factors that discriminate between them. Soc Sci Med.

[B27] Rashley LH (2005). "Work It Out with Your Wife": Gendered Expectations and Parenting Rhetoric Online. NWSA Journal.

[B28] Netmums "Netmum's coffeehouse". http://www.netmums.com.

[B29] Kouri P, Turunen H, Tossavainen K, Saarikoski S (2006). Online discussions mirroring family life during pregnancy. Informatics in Primary Care.

[B30] Valaitis RK, Sword WA (2005). Online Discussions with Pregnant and Parenting Adolescents: Perspectives and Possibilities. Health Promotion Practice.

[B31] Gribble KD (2001). Mother-to-mother support for women breastfeeding in unusual circumstances: a new method for an old model. Breastfeeding Review: Professional Publication of the Nursing Mothers' Association of Australia.

[B32] Russell S (2006). Netmums: online support for parents. Community Practitioner: The Journal of the Community Practitioners' & Health Visitors' Association.

[B33] Sarkadi A, Bremberg S (2005). Socially unbiased parenting support on the Internet: a cross-sectional study of users of a large Swedish parenting website. Child Care Health Dev.

[B34] Fox S (2005). Health information online. Report.

[B35] Graber MA, Roller CM, Kaeble B (1999). Readability levels of patient education material on the World Wide Web. Journal of Family Practice.

[B36] Blackburn C, Read J, Hughes N (2005). Using the Internet? The experiences of parents of disabled children. Child: Care, Health and Development.

[B37] Fox S, Livingston G (2007). Latinos Online: Hispanics with lower levels of education and English proficiency remain largely disconnected from the internet. Report Washington: Pew Internet & American Life Project.

[B38] Brodie MA, Flournoy R, Altman D, Blendon R, Benson J, Rosenbaum M (2000). Health Information, the Internet, and the digital divide. Health affairs.

[B39] Statistics Sweden Privatpersoners användning av datorer och Internet 2007 (Use of computers and the Internet by private persons in 2007). http://www.scb.se.

[B40] Eurostat Internet access and e-skills in the EU27 in 2007. Eurostat news release, 166/2007.

[B41] Berland GK, Elliott MN, Morales LS, Algazy JI, Kravitz RL, Broder MS, Kanouse DE, Muñoz JA, Puyol JA, Lara M, Watkins KE, Yang H, McGlynn EA (2001). Health information on the Internet: accessibility, quality, and readability in English and Spanish. JAMA.

[B42] Mankuta D, Vinker S, Shapira S, Laufer N, Shveiky D (2007). The use of a perinatal internet consultation forum in Israel. BJOG:An International Journal of Obstetrics and Gynaecology.

[B43] Boston M, Ruwe E, Duggins A, Willging JP (2005). Internet use by parents of children undergoing outpatient otolaryngology procedures. Archives of Otolaryngology-Head & Neck Surgery.

[B44] Pandolfini C, Impicciatore P, Bonati M (2000). Parents on the web: risks for quality management of cough in children. Pediatrics.

[B45] Eysenbach G, Powell J, Kuss O, Sa ER (2002). Empirical studies assessing the quality of health information for consumers on the world wide web: a systematic review. JAMA.

[B46] Impicciatore P, Pandolfini C, Casella N, Bonati M (1997). Reliability of health information for the public on the World Wide Web: systematic survey of advice on managing fever in children at home. BMJ.

[B47] Dornan BA, Oermann MH (2006). Evaluation of breastfeeding. Web sites for patient education. MCN Am J Matern Child Nurs.

[B48] Shaikh U, Scott BJ (2005). Extent, accuracy, and credibility of breastfeeding information on the Internet. J Hum Lact.

[B49] Hardwick J, MacKenzie F (2002). Information contained in miscarriage-related websites and the predictive value of website scoring systems. European Journal of Obstretics & Gynecology and Reproductive Biology.

[B50] Ernst E, Schmidt K (2002). Health risks over the Internet: advice offered by "medical herbalists" to a pregnant woman. Wiener Medizinische Wochenschrift.

[B51] Delamothe T (2000). Quality of websites: kitemarking the west wind. BMJ.

[B52] Lewis D, Gunawardena S, El Saadawi G (2005). Caring connection: developing an Internet resource for family caregivers of children with cancer. Comput Inform Nurs.

[B53] Eysenbach G, Köhler C (2002). How do consumers search for and apprise health information on the world wide web? Qualitative study using focus groups, usabillity tests, and in-depth interviews. BMJ.

[B54] HON (2007). Health on the net foundation. http://www.hon.ch.

[B55] Morris SN, Dollahite DC, Hawkins AJ (1999). Virtual Family Life Education: A Qualitative Study of Father Education on the World Wide Web. Family Relations.

[B56] Hudson DB, Campbell-Grossman C, Fleck MO, Elek SM, Shipman A (2003). Effects of the New Fathers Network on first-time fathers' parenting self-efficiency and parenting satisfaction during the transition to parenthood. Issues in Comprehensive Pediatric Nursing.

[B57] Bae J, Heitkemper M (2006). Development of a web-based health information service system for maternal health care. Studies in Health Technology and Informatics.

[B58] Kouri P, Turunen H, Palomaki T (2005). 'Maternity clinic on the net service' and its introduction into practice: experiences of maternity-care professionals. Midwifery.

[B59] Wade SL, Wolfe CR, Pestian JP (2004). A web-based family problem-solving intervention for families of children with traumatic brain injury. Behavior Research Methods, Instruments, & Computers: A Journal of the Psychonomic Society.

[B60] Han HR, Belcher AE (2001). Computer-mediated support group use among parents of children with cancer-an exploratory study. Comput Nurs.

[B61] Nicholas DB, McNeill T, Montgomery G, Stapleford C, McClure M (2003). Communication Features in an Online Group for Fathers of Children with Spina Bifida: Considerations for Group Development among Men. Social Work with Groups.

[B62] Oermann MH, Gerich J, Ostosh L, Zaleski S (2003). Evaluation of asthma websites for patient and parent education. Journal of Pediatric Nursing.

[B63] Hudson DB, Elek SM, Westfall JR, Grabau A, Fleck MO (1999). Young Parents Project: A 21 st-Century Nursing Intervention. Issues in Comprehensive Pediatric Nursing.

[B64] Hsieh Y, Brennan PF (2005). What are pregnant women's information needs and information seeking behaviors prior to their prenatal genetic counseling?. AMIA Annual Symposium Proceedings/AMIA Symposium Provided by American Medical Informatics Association, s.

[B65] Wang HH, Chung UL, Sung MS, Wu SM (2006). Development of a Web-based childbirth education program for vaginal birth after C-section (VBAC) mothers. J Nurs Res.

[B66] Rees T (2002). Web site helps families cope with childhood illnesses. Children's Hospital of Philadelphia undergoes Internet expansion. Profiles Healthc Mark.

[B67] Kildea S, Barclay L, Brodie P (2006). Maternity care in the bush: using the Internet to provide educational resources to isolated practitioners. Rural Remote Health.

[B68] Nyström K, Öhrling K (2006). Parental support: Mothers' experience of electronic encounters. Journal of Telemedicine and Telecare.

[B69] Herman J, Mock K, Blackwell D, Hulsey T (2005). Use of a pregnancy support web site by low-income African American women. Journal of Obstetric, Gynecologic, and Neonatal Nursing: JOGNN/NAACOG.

[B70] Baum LS (2004). Internet parent support groups for primary caregivers of a child with special health care needs. Pediatric Nursing.

[B71] Capitulo KL (2004). Perinatal grief online. MCN the American Journal of Maternal Child Nursing.

[B72] Plantin L (2008). Fatherhood and health outcomes in Europe. http://www.euro.who.int/.

[B73] Bremberg S (2006). New tools for parents Proposals for new forms of parent support Swedish national institute of public health, Report.

